# Molecular species forming at the α-Fe_2_O_3_ nanoparticle–aqueous solution interface†
†Electronic supplementary information (ESI) available. See DOI: 10.1039/c7sc05156e


**DOI:** 10.1039/c7sc05156e

**Published:** 2018-04-20

**Authors:** Hebatallah Ali, Robert Seidel, Marvin N. Pohl, Bernd Winter

**Affiliations:** a Fritz-Haber-Institut der Max-Planck-Gesellschaft , Faradayweg 4-6 , D-14195 Berlin , Germany . Email: winter@fhi-berlin.mpg.de; b Fachbereich Physik , Freie Universität Berlin , Arnimallee 14 , D-14195 Berlin , Germany; c Helmholtz-Zentrum Berlin für Materialien und Energie , Albert-Einstein-Straße 15 , D-12489 Berlin , Germany; d Humboldt-Universität zu Berlin , Department of Chemistry , Brook-Taylor-Str. 2 , D-12489 Berlin , Germany

## Abstract

We report on electronic structure measurements of the interface between hematite nanoparticles (6 nm diameter) and aqueous solutions. Using soft X-ray photoelectron spectroscopy from a liquid microjet we detect valence and core-level photoelectrons as well as Auger electrons from liquid water, from the nanoparticle–water interface, and from the interior of the aqueous-phase nanoparticles. Most noteworthy, the method is shown to be sufficiently sensitive for the detection of adsorbed hydroxyl species, resulting from H_2_O dissociation at the nanoparticle surface in aqueous solution. We obtain signal from surface OH from resonant, non-resonant, and from so-called partial-electron-yield X-ray absorption (PEY-XA) spectra. In addition, we report resonant photoelectron measurements at the iron 2p excitation. The respective Fe iron 2p_3/2_ edge (L_3_-edge) PEY-XA spectra exhibit two main absorption peaks with their energies being sensitive to the chemical environment of the Fe^3+^ ions at the nanoparticle–solution interface. This manifests in the 10*D*_q_ value which is a measure of the ligand-field strength. Furthermore, an observed intensity variation of the pre-peak, when comparing the PEY-XA spectra for different iron Auger-decay channels, can be assigned to different extents of electron delocalization. From the experimental fraction of local *versus* non-local autoionization signals we then find a very fast, approximately 1 fs, charge transfer time from interfacial Fe^3+^ into the environment. The present study, which is complementary to ambient-pressure photoemission studies on solid-electrolyte systems, also highlights the multiple aspects of photoemission that need to be explored for a full characterization of the transition-metal-oxide nanoparticle surface in aqueous phase.

## Abstract 2

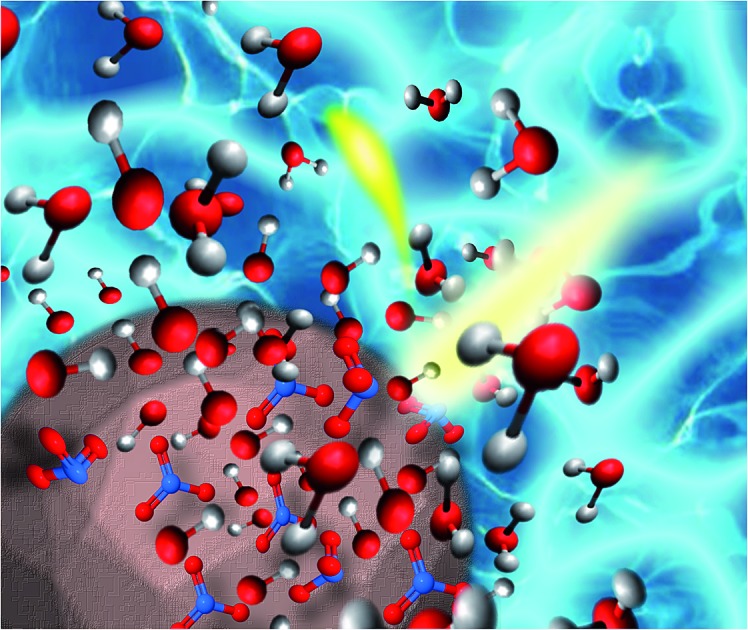
Local electronic-structure interaction, dissociative water adsorption, and electron-delocalization time at the α-Fe_2_O_3_ nanoparticle–aqueous solution interface are revealed from liquid-jet photoelectron spectroscopy at the oxygen-1s and iron-2p resonances.

## Introduction

Iron oxides are highly abundant metal-oxide minerals on Earth^1^ and play a prominent role in many environmental and technological processes,^2^–^6^ relevant for instance in mineralogy and atmospheric science, including corrosion, catalysis, crystal growth and dissolution, as well as photo-electrochemical water splitting. Particularly the latter is of large interest in current energy research, and a central goal is to determine the atomic/molecular and electronic structure of the interface between transition-metal oxide surface and liquid water. Here we focus on photoelectron (PE) spectroscopy to determine the electronic structure.

Experimentally, photoelectron-spectroscopy investigations of the solution–solid interface remain challenging, and so are any other electron-based imaging and spectroscopy techniques routinely used in surface-science studies, requiring considerable adjustment for aqueous phase applications. In recent years several experimental developments have been demonstrated. These are (1) ambient pressure photoelectron (AP-PE) spectroscopy,^7^–^11^ (2) photoelectron spectroscopy from liquid cells consisting of a few layers thick graphene (oxide) membrane with large transmission for electrons in the <500 eV kinetic energy (KE) range,^12^–^14^ and (3) liquid-microjet PE spectroscopy.^15^–^23^ AP-PE spectroscopy detects electrons ejected from the solid surface covered with a few-layer water film, stabilized at approximately 20 mbar water atmosphere. Such near-ambient pressure measurements require experimental conditions that enable collision-free travel of the electrons escaping the solution to the electron analyzer; *i.e.*, long enough electron mean free path must be ensured. The same also applies to the liquid-jet experiments, which are however typically performed at much lower pressure, ∼10^–4^ mbar, and requirements for differential pumping are not as strict. Application of PE spectroscopy, arguably the most important electronic-structure technique, with its unique sensitivity to the atomic chemical environment, then enables detection of the molecular species at the solution–solid interface. The species are identified by their respective electron binding energies (BE), and in some cases by the electronic relaxation processes such as Auger decay or other autoionization channels, which is a central aspect of the present study. One of the main scientific challenges is to explore how exactly water interacts with a solid surface. This includes an understanding of the possible rearrangements of the solid surface structure, connected with catalyzed water dissociation, which would ultimately enable the control of surface properties.

The present work reports on hematite, α-Fe_2_O_3_, nanoparticles (NPs) in aqueous solution. Hematite is the thermodynamically most stable iron oxide, and its interaction with water is promising for photocatalytic (cheap) solar H_2_ production.^24^–^31^ Several experimental^32^–^38^ and theoretical^39^–^42^ studies have been reported for single crystal surfaces. There are likely to exist six possible surface terminations of hematite^39^ which can be classified into two categories, oxygen and iron terminations. Relative stabilities depend on temperature and oxygen pressure during the preparation process of the crystalline surface.^43^ However, a detailed understanding of the hematite termination remains unresolved.^44^,^45^ There is general consensus that gaseous H_2_O dissociates at the hematite surface at both high^46^ and low^47^ vapor exposure. Note that almost all experimental studies have been performed for gas-phase water adsorption in ultra-high vacuum^1^,^46^–^49^ or at ambient pressure.^50^,^51^ Dissociative water adsorption is also found in density functional theory^52^–^55^ and molecular dynamics (MD) simulations.^56^

The electronic structure of the hematite–liquid–water interface has been investigated rather little. On the experimental side we are aware of one single but significant AP-PE spectroscopy measurement from the hematite–liquid–water interface.^57^ It was concluded that H_2_O adsorption on the α-Fe_2_O_3_(0001) surface at near ambient-pressure conditions leads to hydroxylation at very low relative humidity (RH). With increasing RH, the OH coverage increases up to one monolayer, and thereafter H_2_O adsorbs molecularly on top of the hydroxylated surface. Based on measured uptake curves of OH and H_2_O as a function of RH the authors suggest cooperative effects among water molecules that lead to water dissociation. The water-catalyzed dissociation is argued to result from the stabilization of the dissociated state due to the strong hydrogen bond between H_2_O and OH which lowers the kinetic barrier for water dissociation.^57^ Finally, observed small oxygen-1s binding energy shifts of adsorbed OH as a function of water coverage are possible indications of the occurrence of different OH species or α-Fe_2_O_3_(0001) surface reconstruction.^57^ We would also like to point out an MD simulation of hematite NPs in water.^58^ Smaller NPs (1.6 nm) were observed to exhibit larger disorder of the crystalline structure, and also the immediate two water layers are less ordered than for the larger (2.7 nm) particles studied. These results are in accord with a combined vibrational spectroscopy and MD simulations study.^59^

In the present study, we perform liquid-jet PE spectroscopy measurements in conjunction with soft-X-rays from hematite NPs dispersed in aqueous solution. This is the all-in-solution approach to investigate the electronic structure of the Fe_2_O_3_–water interface, and no such attempt has been reported previously. Although we expect that the actual O 1s photoelectron spectrum from dissociated H_2_O at the NP surface will not provide consequential new information with regard to aforementioned AP-PE study from hematite crystal,^57^ there is however an interest in aqueous suspension of hematite NPs for potential (photo)electrochemical applications. We also like to point out that liquid jet studies have the advantage that photon beam damage or impurities (often carbon) encountered in AP studies are essentially absent in flowing samples. Yet, NP (aq) studies remain challenging and complicated for several reasons. One issue is the preparation of aqueous solutions in which the NPs are prevented from aggregation. Another concern is the small electron escape depth in aqueous solution^60^–^62^ which would suggest that detection of electrons with kinetic energies below approximately 500–700 eV (approximately covering the energies of the Auger electrons considered in this work), originating from the NPs in solution, is unfeasible. Regarding the first point preparation of stable NP (aq) suspension requires addition of a stabilizer, typically by pH variation. Only if the surfaces are charged the particles will be electrostatically repelled from each other, and do not form aggregates that sediment out. Inevitably, the adsorption of charged molecules at the NP surface implies that the neat Fe_2_O_3_ NP–water interface would be difficult to explore; this also applies to previously reported aqueous-phase NP studies.^63^,^64^ In the present case α-Fe_2_O_3_ NPs, 6 nm diameter, are stabilized in 0.05 and 0.1 M HNO_3_ aqueous solution, yielding a positive zeta potential. At this acidic pH the NP surface will interact with NO_3_^–^ anions; at these low concentrations all HNO_3_ molecules in the solution dissociate into NO_3_^–^ and H^+^(H_3_O^+^).^65^ It is thus crucial to explore and establish experimental conditions that reasonably balance the stabilizer concentration with a large enough number of NP surface sites for interaction with H_2_O molecules. With respect to the second point, we show here that liquid-jet PE spectroscopy is capable to detect the electronic structure of the hematite NP–aqueous solution interface despite the small electron mean free path in solution.^62^ Specifically, from a combination of core-level and resonant (oxygen 1s and the iron 2p edges) valence PE measurements, and also from analysis of the derived partial-electron-yield X-ray absorption (PEY-XA) spectra we observe the small signal from adsorbed OH species which can be distinguished from the nitrate species, NO_3_^–^ (aq) and NO_3_^–^ (ads). Furthermore, the interfacial electron signal can be distinguished from the electrons emitted from the interior of the aqueous-phase NPs.

## Methods and materials

The photoemission measurements were conducted using the SOL^3^ liquid-jet PE spectroscopy setup^66^ at the U49 PGM soft-X-ray beamline of the synchrotron-radiation facility BESSY II, Berlin. Electrons were detected in a direction perpendicular to the polarization vector of the X-ray beam, with the latter intersecting the horizontal liquid jet also at 90° angle. The liquid jet was produced by pushing the aqueous solution at a flow rate of 1.2 ml min^–1^ and at approximately 25 bar through a 35 μm inner-diameter quartz capillary into the vacuum chamber. This diameter is considerably larger compared to typically 15–20 μm in most of our previous liquid-jet PE spectroscopy studies,^16^,^18^ but the larger size was found here to deliver more stable jets in the case of NP solutions. The jet temperature at the position of interaction with the X-rays (approximately 0.3 mm downstream of the glass capillary) was approximately 2–5 °C. This is a crude estimate, accounting for the reservoir temperature of 10–15 °C and the varying relative amount of measured water gas-phase signal intensity when ionizing the liquid jet further downstream. An exact determination of the jet temperature has been reported for 10 μm diameter based on a measurement of the velocity distribution of evaporating water molecules, yielding a temperature of ∼6 °C.^67^ Under liquid-jet operation conditions a pressure of 7.5 × 10^–4^ mbar was maintained in the interaction chamber using a molecular turbo pump (1600 L s^–1^) and two liquid-nitrogen cold traps. Using an 80 μm exit slit of the beamline the energy resolution at 500 eV photon energy (near the oxygen K-edge) was better than 130 meV, and at 700 eV photon energy (iron L-edge) the resolution was better than 200 meV. The focal size of the X-ray beam was approximately 60 × 60 μm^2^. Electrons were detected with a HiPP-2 (ScientaOmicron) hemispherical energy analyzer which is part of SOL^3^. The 500 μm-diameter detector orifice was at 0.5 mm distance from the liquid jet. With the analyzer pass-energy set to 100 eV the energy resolution was approximately 100 meV in our experiments.

Iron oxide, α-hematite (Fe_2_O_3_), nanoparticles of 6 nm diameter dispersed in 0.05 and 0.1 M HNO_3_ aqueous solutions, were purchased from PlasmaChem [; http://www.plasmachem.com]. The following three NP solutions were studied: 5 wt% NPs in 0.1 M HNO_3_ aqueous solutions (pH 1.55), 10 wt% NPs in 0.1 (pH 1.9) and 0.05 M (pH 2.0) HNO_3_ aqueous solutions. The 0.05 M stabilizer concentration was found to correspond to the smallest amount of stabilizer, NO_3_^–^, at which the NPs stay separated.

## Results and discussion

### Valence photoelectron spectra

**Fig. 1:**
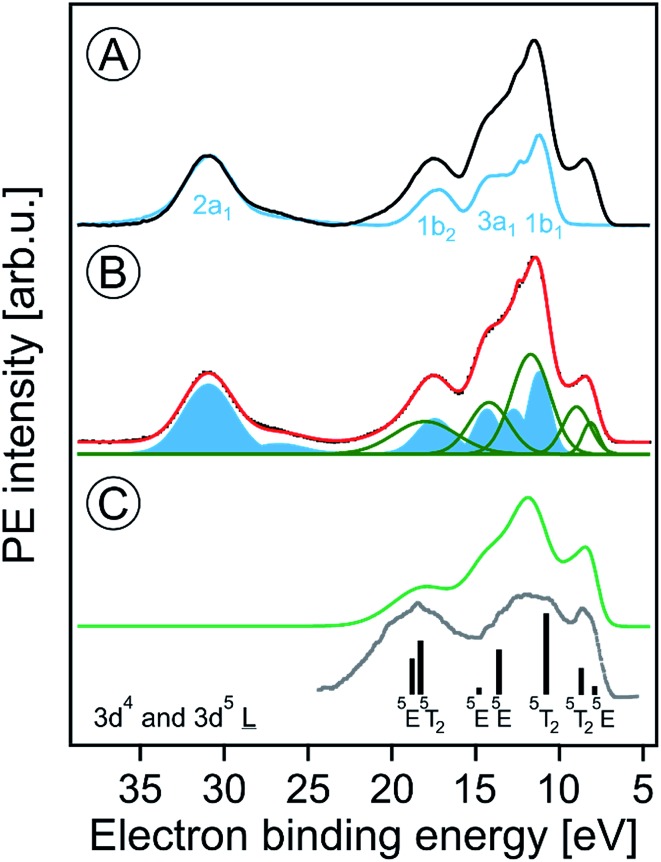
(A) Valence photoelectron spectra from a 5 wt% α-hematite Fe_2_O_3_ NP aqueous solution obtained at the iron 2p_3/2_ resonant photon energy 710.5 eV (black) and at the off-resonant photon energy 704.5 eV (blue). A Shirley background has been subtracted. Contributions from water ionization are labeled. (B) Decomposition of the 710.5 eV spectrum of (A) into contributions from water (blue-filled Gaussians) and iron (green Gaussians). The black-dotted line is the total fit. (C) The green spectrum represents solute-only spectral contributions; it is the sum of the green Gaussians in (B). The grey line is the photoelectron spectrum from solid α-Fe_2_O_3_ measured in ultrahigh vacuum; from ref. 70. The black sticks are calculated energy positions and weights from ref. 74. Both the grey spectrum and the calculated energies were shifted by the work function (5.4 ± 0.2 eV)^46^ as to match the liquid-jet spectra which are presented with reference to the vacuum level.

Fig. 1A presents the valence PE spectra from a 6 nm hematite α-Fe_2_O_3_ NPs, 5 wt%, aqueous solution with added HNO_3_ (0.1 M), measured at photon energies 710.5 (in black) and 704.5 (blue) eV. The former energy is resonant with the lowest-energy Fe 2p_3/2_ → valence excitation, and the latter energy corresponds to off-resonant valence ionization. Spectra shown in the figure are displayed on the binding energy (BE) axis, with energies given relative to the vacuum level.^16^ Both spectra are presented as measured but a Shirley background has been subtracted. Relative intensities of the two spectra are displayed to yield the same height of the water 2a_1_ peak (near 32 eV BE) as this inner-valence peak remains unaffected by the resonant excitation. The spectral energy positions corresponding to ionization of water orbitals 1b_1_, 3a_1_, 1b_2_, and 2a_1_ are labeled.

The most striking observation in Fig. 1A is the absence of the low-energy emission band near 8.5 eV BE for the off-resonant ionization. This immediately illustrates the increased sensitivity of resonant PE (RPE) spectroscopy to otherwise weak photoelectron signals. As we have shown in our previous studies on Fe^3+^ (ref. 68) and Ti^3+^ (ref. 69) aqueous solutions, the direct valence ionization and the (valence → 2p participator) Auger decay produce the same final states. This leads to the coherent superposition of the outgoing electron waves for the two different channels and causes the observed signal enhancement.^68^ Hence the off-resonant PE spectrum in Fig. 1A is essentially the spectrum of neat water, and we can analyze the 710.5 eV RPE spectrum by eliminating the water contributions. For that we first fit the 704.5 eV (off-resonant) spectrum with the known water photoelectron peak positions and widths (determined for the much lower ionization energy of 180 eV (ref. 17)); intensities are kept as free parameters to account for unknown variations of ionization cross sections when increasing the photon energy. The respective water contributions are presented by the blue Gaussians in Fig. 1B. The signal arising from NP ionization is then accounted for by introducing five unique additional Gaussians (green curves); the total fit in Fig. 1B, shown in red, accurately reproduces the 710.5 eV RPE spectrum. In Fig. 1C, we present the spectrum resulting from summing up the green Gaussians in Fig. 1B that represent signal from NP ionization. A detailed interpretation will be provided below with the help of PE spectra measured at the oxygen 1s edge. We note that the experimental NP valence spectrum is in an overall fair agreement with a previously reported valence PE spectrum from crystalline α-hematite^70^ (in gray) measured in ultra-high vacuum. There are clearly distinct differences though. Most noticeable is the occurrence of extra PE signal near 14.5 eV BE, which is due to the effect of the aqueous solution on the Fe 3d–O 2p hybridization which causes a strong ligand-to-iron charge transfer^68^ in this metal oxide.

The major conclusion from Fig. 1A and B is that electron emission from the Fe_2_O_3_ NPs is definitely detectable in the present liquid-jet experiments. This is not self-evident because of aforementioned small electron mean free paths in aqueous solutions at the present KEs;^60^,^62^ this will be detailed later. The lowest-ionization energy peak in Fig. 1B (due to the highest-occupied molecular orbital, HOMO, which is of metal 3d nature) of the solution cannot be fit by a single peak. We assign the two Gaussians, at 8.2 eV and 9 eV BE, to the t_2g_ (with 3-electron occupancy) and e_g_ (2 electrons) levels which arise from the iron 3d^5^ high-spin levels in an octahedral ligand field.^71^ To support this assignment, we need to collect more electronic structure information though, for instance from the PEY-XA spectra which provide the 10*D*_q_ values. Moreover, we must explore whether the observed energies are due to electrons emitted from the NP surface or the interior. It should be noted that the 8.2 and 9 eV energy positions are smaller than the respective iron t_2g_ and e_g_ energies, 8.9 and 10.2 eV, reported for aqueous Fe^3+^ cation.^68^ On the other hand, a single peak at 10.3 eV BE was observed in a later liquid-jet PE study.^72^ Such differences between the iron-oxide NP and the iron hexa–aqua complex can be attributed to the different ligand fields arising from the specific local environments. Previously reported valence PE spectra from the α-Fe_2_O_3_ (1012) crystalline surface in ultra-high vacuum also exhibit a broad unresolved HOMO peak near 8.5 eV,^70^ similar to the spectrum in Fig. 1A. Also, a very recent valence PE spectroscopy study from a powder of 7 nm diameter iron-oxide NPs exhibits a single broad Fe 3d derived peak.^73^ Yet, theoretical calculations^74^ do reproduce the split as shown by the sticks in Fig. 1C.

### O 1s resonant photoemission and partial-electron-yield XA spectra

**Fig. 2:**
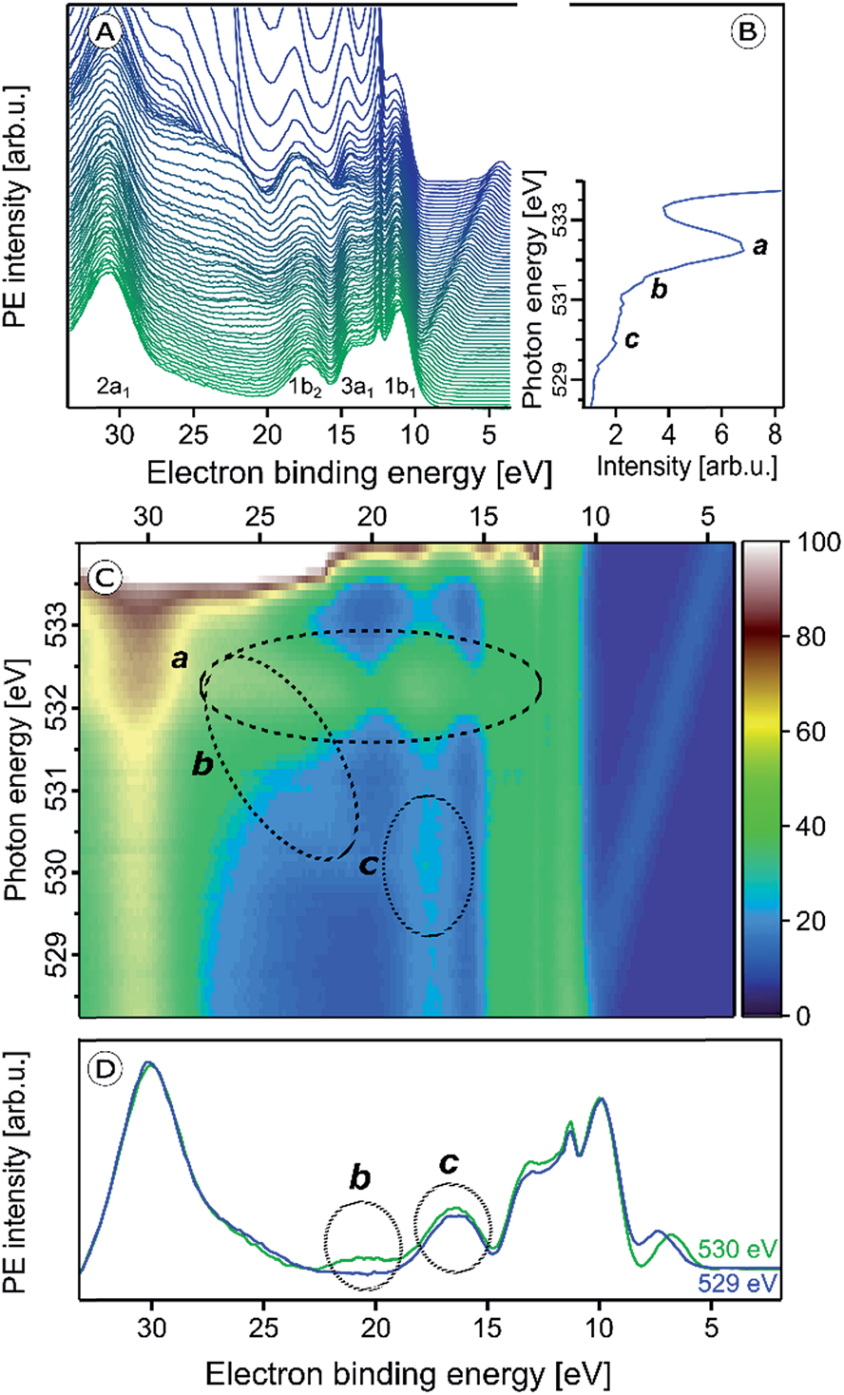
(A) Series of oxygen 1s resonant photoelectron (RPE) spectra from 5 wt% α-Fe_2_O_3_ NPs in 0.1 M HNO_3_ aqueous solution. (B) Resulting partial electron yield X-ray absorption (PEY-XA) spectrum. Peak a (532.2 eV) is the absorption of NO_3_^–^. Bands b (531.5 eV) and c (530.0 eV) are absorptions by lattice oxide of the hematite NPs. (C) Contour map of the oxygen 1s RPE spectra shown in (A); spectral intensities are given by the color code on the right side. Absorptions a–c, are marked by the three black circles. (D) RPE spectrum from Fig. 2A for photon energy 530.0 eV (c resonance) together with the off-resonant valence PE spectrum measured at 529.0 eV. Important to notice is the slightly larger intensity in the 530 eV spectrum at 17.5 eV BE (bottom tier) which is the same contribution that gives rise to the weak signal enhancement, labeled c, in Fig. 2C. More details are provided in Fig. SI-1 of the ESI.†

We now explore the photoemission spectra measured for photon energies near the oxygen 1s core-level ionization threshold. These (valence) RPE spectra contain contributions from direct valence photoionization and from non-radiative relaxation channels, associated with the oxygen 1s core-hole refill. Results from 5 wt% α-Fe_2_O_3_ NPs of 6 nm diameter, dispersed in 0.1 M HNO_3_ aqueous solution (same solution as in Fig. 1), are presented in Fig. 2A. At photon energies <528.0 eV one essentially measures the valence PE spectra from neat water; compare our discussion on Fig. 1A. All spectral changes occurring for increasing photon energies are then associated with the oxygen 1s electron promotion into the partially occupied (O 2p)/(Fe t_2g_, e_g_) hybridized molecular orbitals, and empty valence orbitals. The first absorption peak of neat water (O 1s → 4a_1_; pre-peak) occurs at 535.0 eV photon energy,^75^,^76^ with an absorption onset at approximately 533.0 eV. In order to accurately determine the full absorption spectrum, we look at the respective O 1s PEY-XA spectrum, presented in Fig. 2B, which was obtained by integrating the signal intensity of each RPE spectrum between 15 and 27 eV BE. The steep water absorption onset at 533.0 eV is clearly visible. All other features, at 532.2 eV (peak a), 531.5 eV (small shoulder b), and 530 eV (peak c) must arise from oxygen-containing species other than bulk-phase H_2_O, and these are of particular interest here.

The origin of the absorption peaks a–c can be explained by an analysis of the accompanying changes among the respective RPE spectra. For convenience, we project the RPE spectra in Fig. 2A onto a color-coded 2-dimensional representation. The resulting photon-energy *versus* electron BE map is shown in Fig. 2C. In addition, we present in Fig. 2D the single RPE spectrum measured at resonance c, as this helps to better visualize this small feature. Absorption peak a is caused by an intensity increase near 17 eV BE, and also by a band of at least six overlapping peaks in the 21.0–24.5 eV BE range, as can be seen by the changing color at 532.2 eV excitation photon energy in the encircled area. These energies correspond to spectator Auger electrons from aqueous-phase NO_3_^–^ as has been measured previously for HNO_3_ aqueous solutions.^66^ The PE spectrum at 532.2 eV resonance (peak a) from a 0.5 M HNO_3_ aqueous solution will be shown later, when we determine the spectral contributions from surface-adsorbed species.

**Fig. 3:**
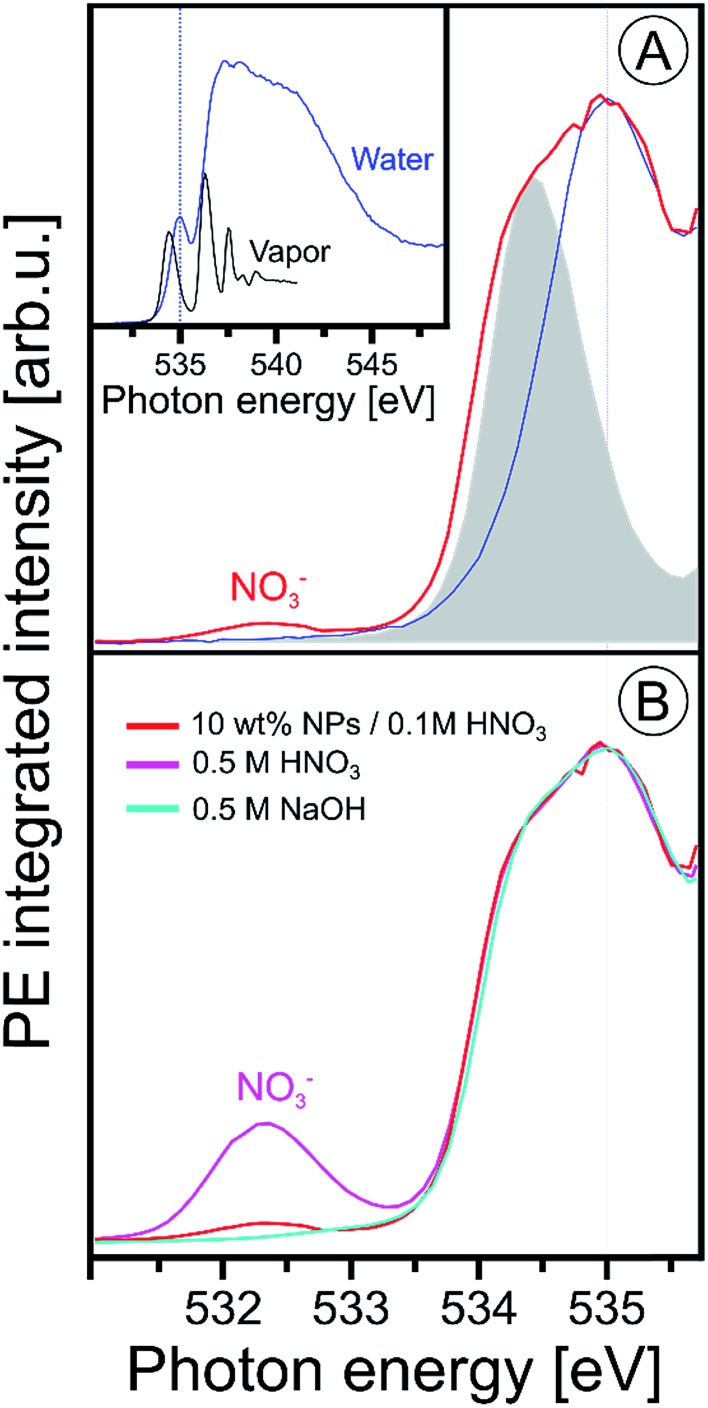
(A) Oxygen 1s PEY-XA spectra from 10 wt% NP/0.1 M HNO_3_ aqueous solution (in red) and from neat water measured under conditions that considerably suppress the spectral contributions from gas-phase water; see discussion in the text. Spectral intensities are adjusted to yield the same heights of the water pre-edge peak, at 535.0 eV. The gray-shaded peak is the leading absorption of gas-phase water; the full spectrum is presented in the inset figure where we also show an extended range of the liquid water spectrum. The latter was obtained from signal integration of the leading Auger peak that overlaps with the valence PE band; the procedure has been discussed in ref. 99. (B) Oxygen 1s PEY-XA spectra from 0.5 M NaOH (light blue), from 0.5 M HNO_3_ (purple), and from 10 wt% NP/0.1 M HNO_3_ aqueous solutions. All spectra in (B) were measured using the same large-diameter glass capillary. Note that under these experimental conditions the absorption peak of hydroxide is not resolved (reported near 533 eV (ref. 90 and 100)), and furthermore the small signal from hydroxide strongly overlaps with the large water gas-phase absorption.

Since the hematite-NP surface is positively charged, as inferred from the measured zeta potential of +30 mV, NO_3_^–^ molecules will inevitably bind at the surface. Evidence for that is indeed found in the O 1s X-ray absorption spectrum, shown in Fig. 3, where we present results from 10 wt% NP/0.1 M HNO_3_, 0.5 M HNO_3_, 0.5 M NaOH aqueous solutions, and from neat liquid water; in addition, the O 1s XA spectrum from gas-phase H_2_O is shown. The liquid water spectrum (blue line), presented in the inset figure, is a PEY-XA spectrum which was previously measured in our laboratory with a smaller capillary (15 μm diameter compared to 35 μm here) to form the liquid jet. Furthermore, in that study a smaller (23 × 12 μm^2^ compared to 60 × 60 μm^2^) X-ray focus was available. Both effects are the reason for a large contribution of gas-phase H_2_O to the aqueous solution XA spectra in the present study, which is clearly seen in Fig. 3A displaying the spectra from the NP solution (in red) and from water (from inset figure), for both gas-phase (gray-shaded peak) and liquid water (blue line) on top of each other. The large shoulder at 534.4 eV observed in Fig. 3A is thus the signal from gas-phase water, and from comparison with the XA spectrum from 0.5 M NaOH (light blue line in Fig. 3B) which exhibits small signal near 533 eV, we see that contributions from gaseous water and OH^–^ (aq) strongly overlap. Since the hydroxide concentration in the NP solutions is so small, a meaningful quantification of its signal on the large water background thus seems unreasonable. Yet, an attempt to single out OH signal based on subtraction of gas-phase signal from the NP (aq) XA spectrum is presented in Fig. SI-2 of the ESI.† It shows measurements from three different NP solutions, each representing a different fraction of surface sites available for H_2_O adsorption. The analysis indeed suggests a small signal from hydroxide at 533 eV, and there is also an indication that the signal intensity may increase with the available surface sites. More details on the data analysis are provided in the ESI.† Unlike the OH^–^ X-ray absorption, NO_3_^–^ absorbs at considerable lower energies than water, near 532.3 eV, as can be seen from the XA spectrum from 0.5 M HNO_3_ aqueous solution in Fig. 3B. Hence, NO_3_^–^ can be easily identified in the NP (aq) XA spectra.

**Fig. 4:**
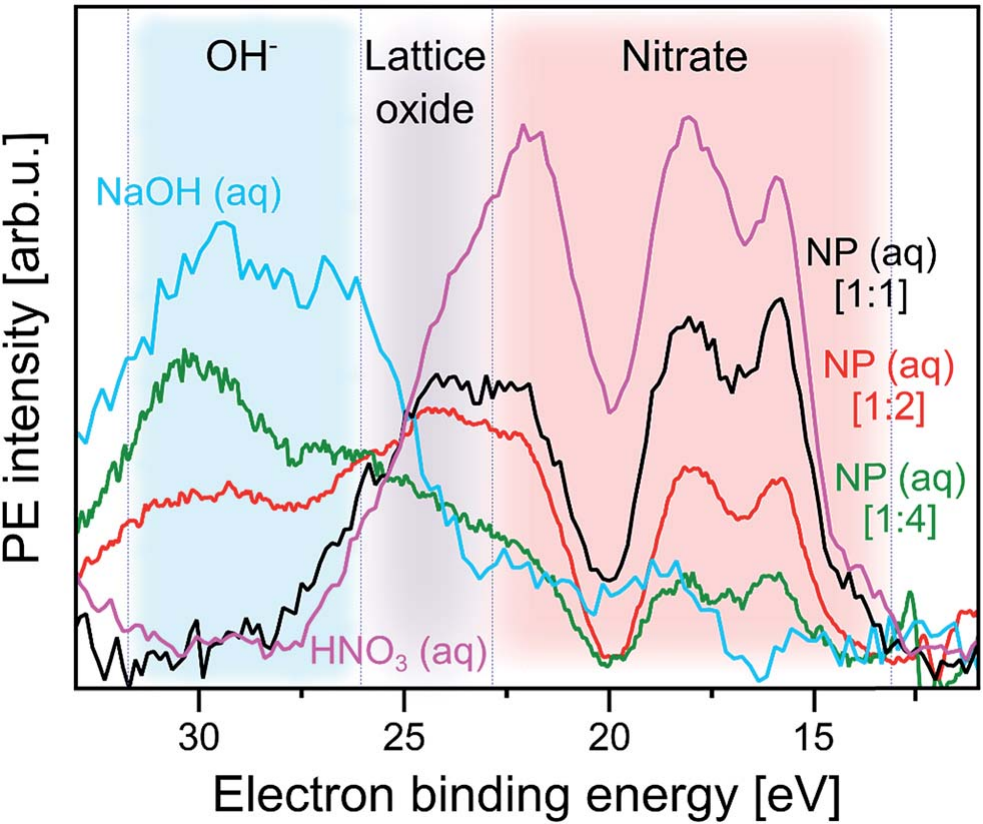
Photoelectron spectra at resonance a (532.2 eV) from three α-Fe_2_O_3_ NPs aqueous solutions, as well as from 0.5 M HNO_3_ and 0.5 M NaOH (measured at 532.8 eV, near a) aqueous solutions. In all cases the respective off-resonant photoelectron spectrum has been subtracted. Results for the following NP solutions are shown: purple: 0.5 M HNO_3_. Black: 5 wt% NP in 0.1 M HNO_3_ [1 : 1]. Red: 10 wt% NP in 0.1 M HNO_3_ [1 : 2]. Green: 10 wt% NP in 0.05 M HNO_3_ [1 : 4]. The additional spectrum from 0.5 M NaOH is shown in blue. The shaded areas mark spectral regions which are dominated by contributions from a single species: blue region: OH^–^. Grey region: lattice oxide. Red region: NO_3_^–^. In square brackets the ratios of number of adsorbed NO_3_^–^ to number of available surface sites of the hematite NPs are shown. These ratios are estimates based on the total surface of the NPs in a given volume, and assuming a density of adsorption sites of 5.6 nm^–2^ which is the value reported for crystalline hematite;^51^ see also description of Fig. SI-2† where we analyze the XA spectra from the same solutions.

In order to obtain a more significant spectroscopic signature from hydroxide adsorbed at the NP–solution interface we next consider the RPE spectra measured at the X-ray absorption maximum, 532.2 eV (peak a; Fig. 2). These spectra are barely affected by gas-phase water, and we can detect adsorbed hydroxide and nitrate simultaneously. Results are shown in Fig. 4 for the same solutions briefly mentioned in the previous paragraph where we discussed the XA spectra. As introduced along with Fig. SI-2† the NPs in the different solutions differ by the amount of adsorbed nitrate, and hence the fraction of available adsorption sites for water varies, which is controlled by the concentrations of (HNO_3_) stabilizer relative to the NPs in the solutions. The nitrate-to-free surface sites ratios studied here are approximately 1 : 1 (labeled [1 : 1] in Fig. 4) for 5 wt% NP in 0.1 M HNO_3_, 1 : 2 ([1 : 2]) for 10 wt% NP in 0.1 M HNO_3_, and 1 : 4 ([1 : 4]) for 10 wt% NP in 0.05 M HNO_3_. For reference, Fig. 4 also includes the spectra from 0.5 M HNO_3_ and 0.5 M NaOH aqueous solutions, the latter is measured at 532.8 eV excitation energy (close to peak a). All spectra are displayed with the corresponding off-resonant spectra subtracted which singles out the spectral features that get enhanced. Note that subtraction of the large water signal is the reason for the rather poor signal statistics. As-measured RPE spectra are shown in Fig. SI-3 of the ESI.† One important observation from Fig. 4 is that the RPE spectrum for highest NO_3_^–^ (ads) concentration (ratio 1 : 1) is almost the same as the one from bare HNO_3_ aqueous solution. In both cases four main photoemission bands are observed, at approximately 16.0, 18.0, 22.5, and 24.5 eV BE (all within the red-shaded area), arising from various Auger-electron decays upon O 1s → valence excitation at 532.2 eV photon energy. The fact that the spectra present close resemblance indicates that the electronic structure of NO_3_^–^ (aq) changes little upon adsorption at the NP surface. The 4-peak structure of the HNO_3_ (aq) spectrum can be qualitatively attributed to Auger processes that involve orbitals with NO_3_^–^ character. These have energies of approximately 9.5, 16.0, 19.5 eV BE, as determined from an (off-resonant; 200 eV photon energy) valence PE spectrum from 1 M HNO_3_ aqueous solution presented in Fig. SI-4.† The leading peak at 15.5 eV is due to a participator Auger decay. A more accurate assignment of the NO_3_^–^ Auger-electron spectrum would require quantification of screening effects of the core hole, and also consideration of the nitrogen *versus* oxygen characters of the orbitals.^77^

With increasing number of available H_2_O adsorption sites on the NP surface (ratio 1 : 4) the RPE spectrum (green in Fig. 4) still exhibits a similar overall shape as the one from NO_3_^–^ (aq) but relative peak intensities in the 15–20 eV BE range change, and peak energies tend to shift slightly. In addition, peaks seem to broaden but this effect cannot be quantified due to insufficient signal statistics. These changes are attributed to the occurrence of signal from adsorbed OH which is concluded from comparison with the 0.5 M NaOH aqueous solution. The respective RPE spectrum (in blue) exhibits a rather similar overall shape as the one from the 1 : 4 NP solution (green). We particularly point out the appearance of a 28.5 eV BE peak (blue-shaded area) for the latter solution which is an unequivocal signature of OH (compare blue spectrum). Occurring differences of all spectral intensities are argued to arise primarily from the varying relative ratios of adsorbed NO_3_^–^ over OH. But we also expect small energy differences between free and adsorbed hydroxide, and we also note that in the NP spectrum the 28.5 eV OH peak overlaps with the electron emission from NP lattice oxygen (23–28 eV BE region; grey-shaded), although this signal has larger intensity at the slightly lower absorption energy corresponding to peak b (531.5 eV) in Fig. 2C.

We return to Fig. 2 to discuss X-ray absorption peaks b and c. Both oxygen-1s excitations must arise from the Fe_2_O_3_ NPs (aq), and we will now differentiate between the contributions from the NP–solution interface *versus* those from the NP interior (bulk). As seen from Fig. 2C absorption b (see also Fig. SI-1†) correlates with a signal increase in the RPE spectra near 23 eV BE, whereas c correlates with a very small increase of the 17.5 eV BE peak. In an attempt to somewhat enhance the visibility of absorption c, we have displayed in Fig. 2D the relevant single RPE spectrum, at 530.0 eV excitation energy, selected from Fig. 2A. This spectrum is compared with the off-resonant PE spectrum measured at 529.0 eV, and one notices a small signal increase at the 17.5 eV BE. In Fig. SI-1 of the ESI† we show that this increase vanishes when the photon energy is raised slightly above the c resonance.

From comparison with L-edge X-ray absorption spectra from hematite crystal measured at ∼10^–4^ mbar water pressure,^78^ we assign absorptions b and c to the O 1s → t_2g_ (at 530.0 eV) and O 1s → e_g_ (at 531.5 eV) transitions, respectively. The energy difference yields 10*D*_q_ = 1.5 eV. Previous XA spectroscopy studies from α-Fe_2_O_3_ (ref. 48) have reported very similar absorption energies of 530.2 and 531.6 eV, respectively, *i.e.*, 10*D*_q_ = 1.4 eV.^49^,^79^–^84^ Another study, of supported 8 and 30 nm hematite NPs^85^ finds absorption maxima at 530.4 (for O 2p–Fe t_2g_) and 531.8 eV (for O 2p–Fe e_g_) photon energies. In the same work, also crystalline hematite has been investigated, and the authors observed the identical 10*D*_q_ value of 1.38 eV, concluding that there is no evidence for a size-driven effect. The slightly larger 10*D*_q_ (1.5 eV) in the present study is thus argued to result from the hematite NP's modified electronic structure in the presence of an aqueous solution. This conclusion will be corroborated by our RPE measurements at the iron 2p_3/2_ (L_3_) edge. With above assignments to electron promotion into t_2g_ and e_g_ states, which are separated by 1.5 eV, one would expect similar RPE spectra for b and c excitations. But this is not observed experimentally. Referring to the computed electronic states of (FeO_6_)^9–^,^48^,^71^ and considering solely involvement of t_2g_ states the peak at 17.5 eV BE (corresponding to ∼512 eV kinetic energy) could be qualitatively understood as arising from O^2–^ 1s-1t_2g_1t_2g_ spectator Auger decay. Here, excitation from the oxygen 1s core-level into the empty 2t_2g_ (spin down) level is followed by core-hole refill from the 1t_2g_ level, and electron release from 1t_2g_. Similarly, for the O^2–^ 1s → 4e_g_ excitation at 531.5 eV the peak at ∼22.5 eV BE (corresponding to ∼509 eV kinetic energy) can be explained by O^2–^ 1s-3e_g_3e_g_ spectator Auger decay. We are unable though to provide a more quantitative explanation based on the available data.

### O 1s core-level photoelectron spectra

**Fig. 5:**
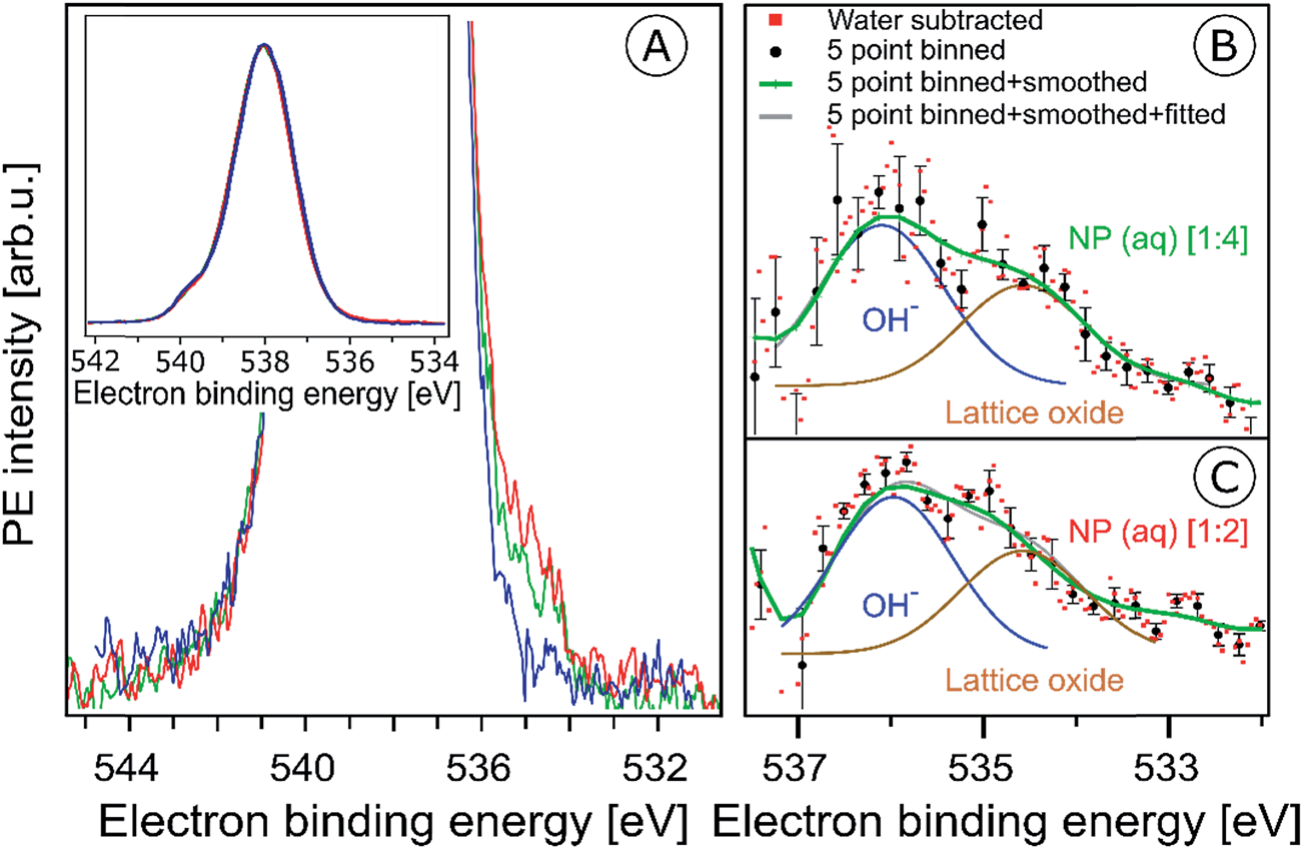
(A) Oxygen 1s photoelectron spectra from the 10 wt% hematite NPs in 0.1 M HNO_3_ aqueous solution (corresponding to [1 : 2]; red curve), and in 0.05 M HNO_3_ aqueous solution (corresponding to [1 : 4]; green curve). For reference, the spectrum from 0.05 M NaCl aqueous solution (blue curve) is also shown. Photon energy was 1200 eV. In the main figure, the O 1s peak has been cut at about 25% of its intensity. The full peaks, and the intensity normalization is seen in the inset figure. (B and C) Oxygen 1s photoelectron spectra in the 538–532 eV binding energy region which covers the O 1s photoelectron peaks from OH^–^ (536.1 eV) and lattice oxide (534.7 eV). (B) shows results for the 10 wt% hematite NPs/0.05 M HNO_3_ aqueous solution (corresponding to [1 : 4]), and (C) for 10 wt% hematite NPs/0.1 M HNO_3_ aqueous solution (corresponding to [1 : 2]) after subtraction of the 0.05 M NaCl aqueous solution spectrum (red dots). Black dots in (B) and (C) result from five-point-binning of the red dots, and the green line results from additional smoothing. Error bars, representing the standard deviation from five-point-binning, are still fairly large. They are too large to reveal the expected increase in the OH-to-lattice oxide signal ratio when going from [1 : 2] to [1 : 4] solution. See also Fig. SI-5† for the raw data.

In the previous section surface bound NO_3_^–^ and OH species were shown to give rise to characteristic signals in the O 1s RPE valence spectra. We now explore the signature of these species in the non-resonant O 1s PE spectra, shown in Fig. 5A. The spectra were obtained from the 6 nm diameter Fe_2_O_3_ NPs (10 wt% aqueous solutions) for the two HNO_3_ concentrations 0.1 M (in red) and 0.05 M (in green). For comparison, the spectrum from 0.05 M NaCl aqueous solution containing no NPs, and essentially representing neat water, is presented as well (in blue). The spectra are normalized to their peak maxima, as shown in the inset of Fig. 5A. A photon energy of 1200 eV was used to deliberately generate O 1s photoelectrons with approximately 650 eV KE, which is similar to the electron energies detected in case of the Fe 2p RPE spectra shown in Fig. 1. This assures a comparable probing depth into the solution for both cases. And more important, the inelastic mean free path of the ∼700 eV photoelectrons is obviously large enough to detect electrons born at the NP (aq) surface and even originating inside the NPs (aq). However, the exact probing depth into the particles as well as the distance of the NPs from the solution surface cannot be quantified here. For recent estimates of electron mean free paths in liquid water and aqueous solutions we refer to ref. 60 and 61.

PE spectra from the two NP solutions (Fig. 5A) are seen to be by far dominated by photoelectrons from liquid water, giving rise to the strong peak at 538.0 eV BE.^86^ A small shoulder at 540.0 eV BE arises from ionization of gas-phase water. The photoelectron signal contributions from oxygen species, primarily OH bound to the NP (aq), appear at lower BE than water, and the intensity is very small, slightly greater than the baseline signal. An enlarged view of the spectral region of interest (with the water spectrum subtracted) is presented in Fig. SI-5A (NP/0.1 M HNO_3_) and SI-5B† (NP/0.05 M HNO_3_). These spectra exhibit very poor statistics though, and in order to demonstrate that the data are yet statistically significant the as-measured individual data points (of Fig. SI-5†) were binned and the resulting error bars have been determined. Results for five-point binning are presented in Fig. 5B and C, where we also provide error bars and Gaussian fits to reproduce the observed double-peak spectrum. A more detailed description of the data handling is given in the caption of Fig. 5. The higher-BE peak at 536.1 eV (1.0 eV width), which is 1.9 eV smaller BE than liquid water, can be assigned to adsorbed OH, in agreement with several reported surface hydroxyl species formed on the hematite crystal surface.^1^,^44^,^87^,^88^ Note that NO_3_^–^ can be ruled out because its O 1s signal contributes to the O 1s (aq) peak at 538.1 eV and cannot be deconvoluted due to the high concentration of bulk water.^63^,^89^ Our assignment also agrees with a previous liquid-jet PE measurement from 4 molal NaOH aqueous solution, reporting a 536.0 eV O 1s BE of OH^–^ (aq).^90^ As in the case of the O 1s RPE spectra (Fig. 4), a distinction between aqueous-phase OH^–^ and the adsorbed hydroxide is not possible on the grounds of the PE spectra of Fig. 5. However, our assignment of adsorbed OH is justified by the acidic pH of the NP solutions. Perhaps another indication is the slight increase of OH signal for the [1 : 4] solution in qualitative agreement with the resonant O 1s photoelectron spectra of Fig. 4. The lower-BE peak at 534.7 eV (*i.e.*, 3.3 eV smaller BE than liquid water) and approximately 1 eV width is attributed to the O 1s BE of the lattice oxygen of the hematite NPs (aq). This is in agreement with the energy difference found in an ambient-pressure PE study of a few-monolayer liquid water film on top crystalline α-Fe_2_O_3_ (0001).^57^ A remaining and somewhat puzzling observation from Fig. 5B and C is that the O 1s signal intensities from the lattice oxide and from surface OH are rather similar. Our explanation is that at the electron kinetic energies relevant here the electron inelastic mean free path approximately matches the distance between NP surface and the solution surface. This is a reasonable assumption, based on reported estimates of the mean free path in aqueous solutions^62^ which strongly encourages liquid-jet PE spectroscopy studies with tender X-rays to probe deeper into both the solution and into the NPs.

### Fe 2p photoemission and PEY-XA spectra

**Fig. 6:**
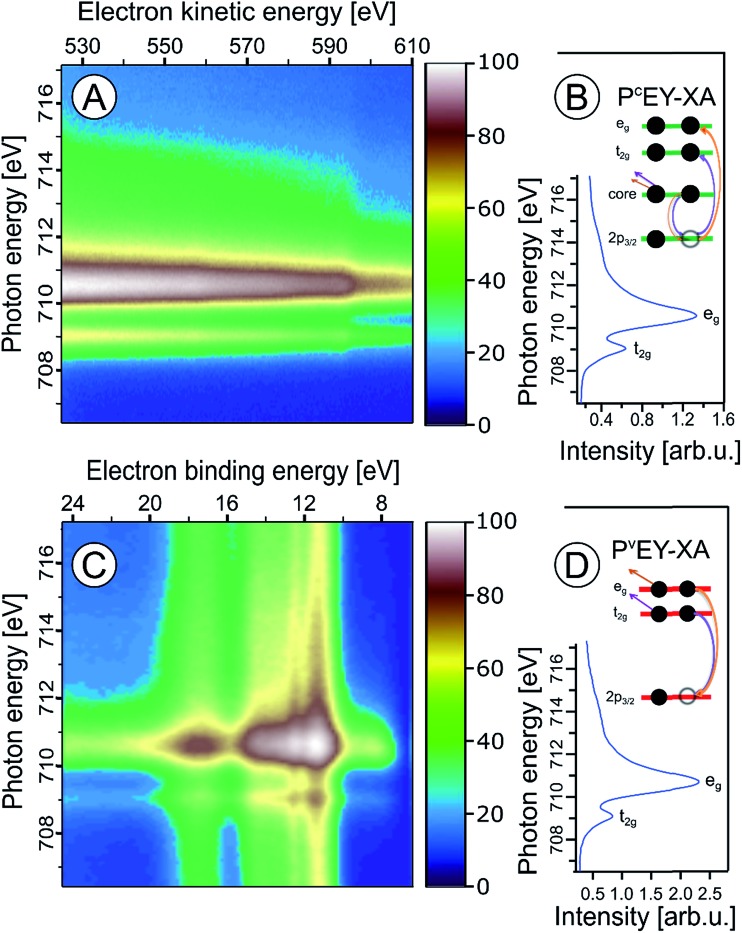
Fe 2p_3/2_ resonant photoelectron spectra from 10 wt% α-Fe_2_O_3_ NPs in 0.1 M HNO_3_ aqueous solution, covering the 2p-3p3p (A) and 2p-3d3d (C) Auger-electron emissions after Fe 2p_3/2_ → t_2g_ and 2p_3/2_ → e_g_ excitation, respectively. Spectra were recorded for photon energies between 707 and 716 eV. The respective P^C^EY- and P^V^EY-XA spectra, and illustrations of the relevant energies, excitations and the relaxation channels are displayed in (B) and (D).

In the following we explore the photoemission spectra for excitation energies resonant with the Fe 2p → valence transitions. The idea of these measurements is analogous to the oxygen 1s excitation discussed along with Fig. 2. We are thus interested in the evolution of the RPE spectra when varying the photon energy across the Fe 2p (only 2p_3/2_, *i.e.*, the L_3_ edge is considered here) resonance, and how this reflects in the Fe L_3_-edge PEY-XA spectra. XA spectra will be obtained for two different relaxation channels, one corresponding to the refill of the 2p core-hole by a valence electron, and in the other case the refill is by another core-level electron. The specific Auger decay channels are 2p-3d3d and 2p-3p3p which lead to electron emission in the 672–715 eV and 560–600 eV kinetic energy ranges, respectively. Hence, signal integration of the RPE spectra within these boundaries yields the respective PEY-XA spectra. In (2p-3d3d) PEY the electron refilling the core hole originates from an iron 3d valence orbital (spectra are denoted as P^V^EY). The 3d orbitals carry information on the mixing with ligand-centered orbitals. On the other hand, in (2p-3p3p) PEY the core-hole is refilled by electrons from the deeper Fe 3p orbitals which only weakly interact with the ligands (spectra are denoted as P^C^EY). Here we use the same nomenclature as in our previous study from FeCl_2_ aqueous solutions.^91^ Illustrations of the two Auger processes will be presented in Fig. 6.

Fig. 6A and C present Fe 2p RPE maps from 10 wt% α-Fe_2_O_3_ NPs in 0.1 M HNO_3_ aqueous solution, covering the 2p-3p3p and 2p-3d3d Auger decay channels; individual spectra were recorded for photon energies between 707 and 717 eV. Fig. 6B and D display the respective P^C^EY- and P^V^EY-XA spectra. Note that Fig. 6A displays data as KEs while Fig. 6C presents BEs in the 5–25 eV range energy, which corresponds to 682–712 eV KE. Valence spectra in Fig. 6C are dominated by an off-resonant water-signal background, appearing as vertical bands. The spectra in Fig. 6A have no off-resonant signal contribution, and solely exhibit resonant signals from 2p-3p3p and in part from 2p-3s3d Auger emissions. Therefore, in the latter case the KE axis is more appropriate since Auger electrons do not depend on the photon energy. Both PEY-XA spectra exhibit a pre-peak at 709 eV photon energy, and a main peak at 710.5 eV. These are the Fe 2p_3/2_ → t_2g_ and Fe 2p_3/2_ → e_g_ resonances characteristic for Fe^3+^.

**Fig. 7:**
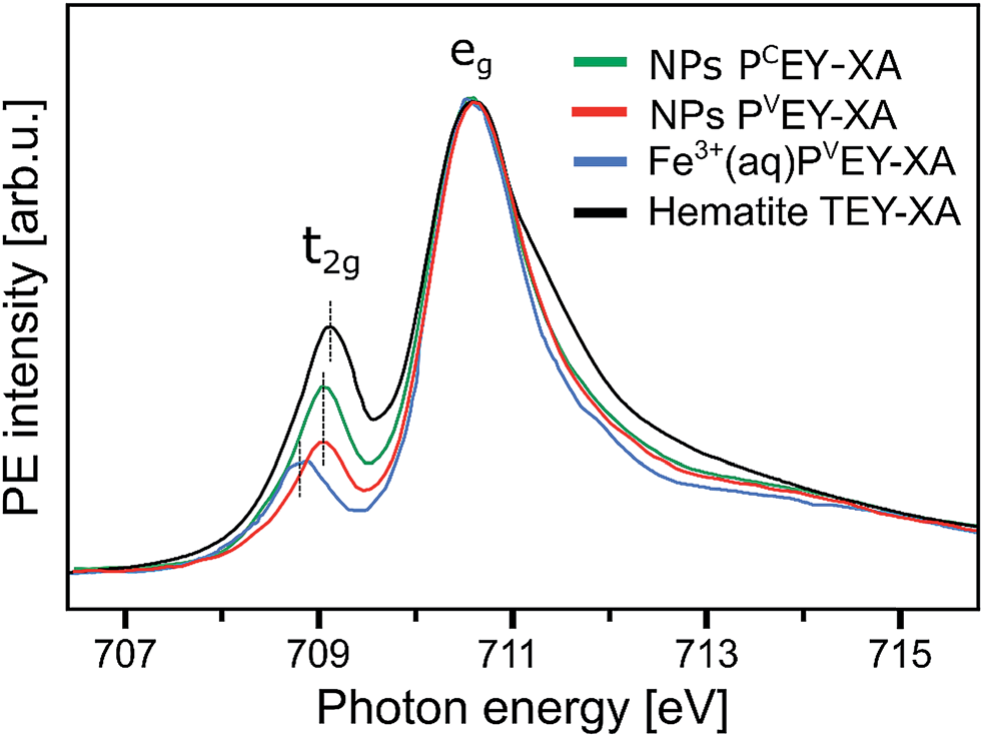
Comparison of the Fe L_3_-edge P^C^EY- and P^V^EY-XA spectra (in green and red) from 10 wt% α-Fe_2_O_3_ NPs in 0.1 M HNO_3_ aqueous solution (of Fig. 6) with the Fe L_3_-edge P^V^EY-XA spectrum from 1 M FeCl_3_ aqueous solution (in blue),^68^ and with the total-electron-yield spectrum from solid hematite sample (in black).^49^ Spectra are displayed to yield the same height of the most intense absorption band, at 710.5 eV photon energy.

**Fig. 8:**
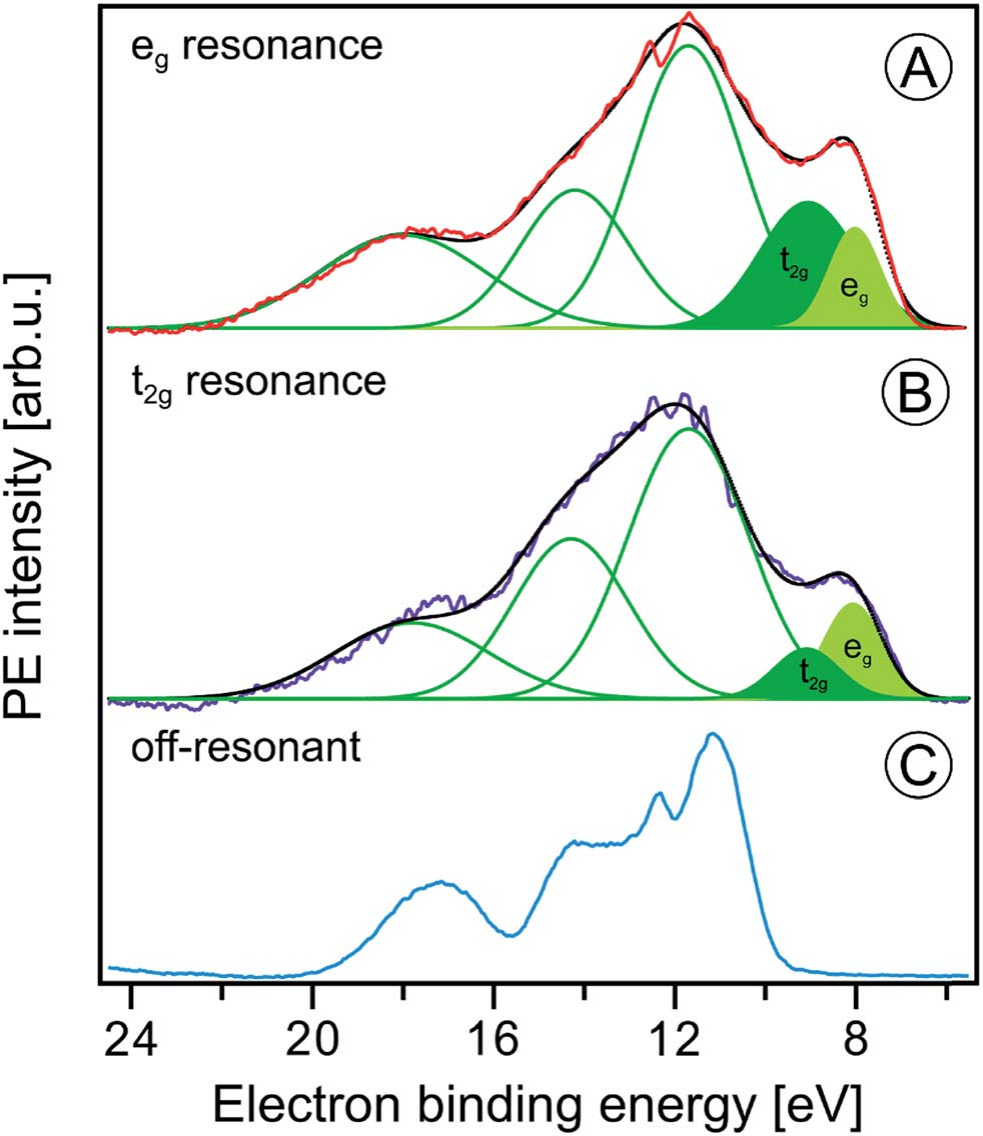
Fe 2p_3/2_ resonant photoelectron spectra from 10 wt% α-Fe_2_O_3_ NPs in 0.1 M HNO_3_ aqueous solution measured at the e_g_ (710.5 eV) and the t_2g_ (709.0 eV) resonances, (A) and (B), respectively. In both cases the off-resonant photoelectron spectrum obtained at 706.0 eV photon energy, shown in (C), has been subtracted. Green curves in (A) and (B) are the Gaussians that represent the spectral contributions from the NPs; compare Fig. 1B.

Fig. 7 presents the P^V^EY- and P^C^EY-XA spectra on top of each other, and one observes a considerably smaller pre-peak in the P^V^EY-XA spectrum; intensities are displayed such that the e_g_ peaks have the same height (to be discussed later). In addition to the different intensities of the pre-peaks, one notices a somewhat smaller intensity in the post-edge region at 709–714 eV. The pre-peak arises from the electronic interactions between the iron site and the local environment, *i.e.*, with the solvation-shell water molecules and the lattice oxygen atoms of the hematite NP. The excited electron can thus engage in additional relaxation processes such as electron delocalization and energy transfer.^91^,^92^ As a consequence, the P^V^EY-XA spectrum may differ considerably from the P^C^EY-XA spectrum which is in fact a better representation of the true X-ray absorption spectrum that would be obtained in a transmission measurement. The delocalized excited electron has a lower probability to refill the Fe 2p hole within its lifetime (sub-10 fs), and hence the PEY is state-dependent, scaling with the extent of delocalization. The observed differences between the P^V^EY- and P^C^EY-XA spectra (Fig. 7) thus correlate with the orbital extensions.^92^ Core-level 3p orbitals are strongly localized and are not or barely involved in iron–ligand orbital mixing. In contrast, 3d orbitals are large, and there is considerable mixing with water lone-pair orbitals as well as with the NP lattice oxygen-2p orbitals which was already seen from Fig. 1. Our interpretation is corroborated by the changes that occur in the RPE spectra measured at the e_g_*versus* the t_2g_ resonance. These two RPE spectra are shown in Fig. 8. In both cases the off-resonant valence PE spectrum, measured at 707 eV (bottom tier in Fig. 8), has been subtracted. To quantify the spectral changes that occur for the two resonances we fit the spectra (center and top tiers in Fig. 8) using the same Gaussians as in Fig. 1B, *i.e.*, energy positions and peak widths are kept constant, and the only free fit parameter is the intensity. The most noticeable observation is the decrease of the t_2g_ signal intensity relative to the e_g_ signal which is exactly what one expects. Due to the overlap of the metal t_2g_ levels with the O 2p levels of hematite (already seen in Fig. 1C) the electron excited into t_2g_ appears to relax by a different path, thus quenching the 2p-3d3d Auger channel. On the other hand, when populating the e_g_ states, which have less overlap with the oxygen orbitals, the excited electron promptly refills the Fe 2p hole *via* the 2p-3d3d Auger channel. These qualitative considerations are in agreement with theoretical calculations, reporting for bulk hematite stronger hybridization between Fe t_2g_ and O 2p orbitals than between e_g_ and O 2p, 48% *versus* 35% oxygen character.^71^

We can go a step further and quantify the observed electron delocalization to estimate the charge transfer, or electron delocalization rate. Fast charge transfer, which inhibits the charge recombination at the surface (for instance of a transition-metal-oxide electrode), is crucial for efficient device performance under photoelectrochemical conditions. With the Fe 2p_3/2_ lifetime, *τ*_core_, assuming an exponential Auger decay rate, and an exponential electron delocalization governed by the charge transfer time, *τ*_CT_, the latter can be expressed as *τ*_CT_ = *τ*_core_(*f*_Auger_^–1^ – 1).^93^,^94^ Here, *f*_Auger_ is the fraction of normal Auger-electron signal with respect to the signal from non-local decay processes, the charge-transfer channels of autoionization. *τ*_core_ = 1.6 fs (1.8 fs) as calculated from the natural line width of Fe 2p_3/2_, *Γ* = 0.41 eV (ref. 95) (0.36 eV (ref. 96)). We determine the normal Auger fraction from the local and non-local signal contributions. Disentangling these signals from Fig. 8 is not straight-forward because both e_g_ and t_2g_ mix with ligand orbitals, and hence there is no obvious way of scaling the relative spectral intensities. Arguably, the more accurate procedure is to use the peak areas of the P^C^EY- and P^V^EY-XA spectra of Fig. 7. In this case, assuming that the intensity normalization at the e_g_ peak is reasonable, the difference in the pre-peak intensity of the t_2g_ absorption does scale quantitatively with the electron delocalization. To be more accurate, we argue that the P^C^EY-XA spectrum is a very good representation of an actual X-ray absorption spectrum.^91^,^92^ Any difference between P^C^EY- and P^V^EY-XA spectra must thus be due to delocalization of the 3d electrons. From the areas of the t_2g_ XA peaks we then find *f*_Auger_ = 0.6 which yields *τ*_CT_ ∼ 1 fs. We are not aware of a previous report of this quantity which would be very difficult to access by other experimental techniques, requiring sub-femtosecond laser pulses.

A question that arises is how 10*D*_q_ (the e_g_–t_2g_ energy difference) from NPs (aq) (see Fig. 7) compares with values for atomic Fe^3+^ in aqueous solution (blue) and bulk-solid-phase hematite (black) under high-vacuum conditions; the XA spectra for the latter are also presented in Fig. 7. In all cases the iron charge-state is Fe^3+^. For nanoparticles in aqueous solution we derive from Fig. 7 a 10*D*_q_ value of 1.5 eV (the same is obtained for the O 1s edge XAS) which is slightly larger than for crystalline hematite, 10*D*_q_ = 1.38 eV,^85^ but it is smaller than for the Fe^3+^ atomic ions in aqueous solution, where 10*D*_q_ = 1.8 eV. A quantitative interpretation of the different values would be difficult but we argue that the main effect is due to the NP–solution interface. This is because aforementioned previous O 1s absorption studies have found an identical 10*D*_q_ value (1.38 eV) for bulk-solid hematite and for dry 8 and 30 nm diameter hematite NPs.^85^ Hence, the smaller 10*D*_q_ is a property of the surface of the aqueous-phase NPs. The Fe 2p_3/2_ PEY-XA spectra are thus sensitive to the interfacial structure, and yet the quantitative characterization of the ligand field, without a good understanding of the surface structure and the existing molecular species, is elusive. A similar qualitative argument probably holds to explain the intensity variation in the 711–714 eV photon-energy region (Fig. 7) – the spectral fingerprint region of excited-state charge transfer.^97^ Intensities follow the same trend as the t_2g_ absorption intensity, although the effect is negligibly small when comparing P^V^EY compared to P^C^EY. The latter would be expected if we assume that the ground-state iron t_2g_–oxygen 2p orbital overlap is only little affected by the electron excited into a higher lying state. This assumption also justifies aforementioned normalization of the spectra at the 2p → e_g_ absorption in Fig. 7. The observed considerably larger intensity, near 712 eV absorption energy, for crystalline hematite compared with the NP solutions, and even more so Fe^3+^ (aq), suggests that ground-state charge transfer from the ligand to the iron cation is smaller in solution, implying less orbital overlap. Arguably, water or hydroxide, either absorbed at the nanoparticle surface or located within the first hydration shell in case of the Fe^3+^ (aq) monomer, have a smaller charge-transfer probability compared to O^2–^ ligands in the bulk hematite. To confirm this interpretation theoretical calculations are needed to quantify charge transfer, including electron donation and back-donation between the different electronic ground-state configurations.

## Conclusions

We have demonstrated that liquid-jet soft-X-ray PE spectroscopy is a powerful method that enables the detailed investigation of the electronic-structure interaction of hematite nanoparticles with an aqueous solution. This is a remarkable result because of the rather short probing depth of the emitted (photo)electrons in aqueous solutions. An experimental challenge of the present work has been to stabilize hematite NPs at sufficiently large concentration with an as small as possible number of stabilizing molecules adsorbed at the NP surface. Using a combination of soft-X-ray photoemission techniques (direct and resonant ionization, and autoionization) electrons from both the surface and the interior of the aqueous-phase NPs can be detected. From the oxygen 1s PE spectra we obtain the electron binding energy from dissociated water, at 536.1 eV, which is in good agreement with the energies of hydroxyl species reported in an ambient-pressure PE-spectroscopy study of the Fe_2_O_3_(0001) hematite–liquid water interface.^57^ However, the new spectroscopic information from our NP study is revealed from resonant PE spectra at the oxygen 1s and iron 2p_3/2_ edges. The former spectra provide a complementary and very sensitive electronic structure signature of oxygen-containing molecules adsorbed at the NP surface. Performing photoemission measurements at the iron 2p_3/2_ edge we were able to detect the lowest ionization energy of the solution, which corresponds to the ionization of the iron 3d-derived e_g_ and t_2g_ orbitals. This is an important quantity for understanding chemical reactions in aqueous solution. We also determined the e_g_–t_2g_ energy difference (10*D*_q_ value) from the partial-electron yield iron 2p_3/2_ XA spectra obtained from an analysis of the Auger electron signal. 10*D*_q_ is a measure of the Fe^3+^ local environment, which is found here to be uniquely sensitive to the iron interactions with both hematite oxygen and water/oxygen interfacial species. Unfortunately, we cannot provide a quantitative interpretation of the energy shifts. But we expect that our experimental findings will motivate theoretical modeling of the rather complex hematite–water interface, taking into account iron spin interactions. An aspect related to the iron 2p RPE measurements is the possibility to obtain (PEY) XA spectra for different autoionization channels which can be used to estimate the ultrafast electron delocalization times of electrons excited into metal 3d orbitals.

One promising future experimental route is the application of tender X-rays in liquid-jet photoemission which allows probing deeper into solution and into the NPs (aq). This will greatly enhance the signal intensity detected from the NP–solution interface, enabling further characterization of the dissociated water species and their interactions with the NPs. With regard to the latter point it will be also interesting to explore non-local relaxation processes upon water O 1s ionization with their large sensitivity to hydration structure and hydrogen-bond strength.^98^ The presented liquid-jet studies demonstrate promising applicability for the investigation of the electronic structure of other NPs, including transition-metal-oxide, also noble metals and other materials, dispersed in various electrolyte solutions over a large range of concentrations.

## Author contributions

H. A., R. S. and B. W. planned the experiment and selected the samples. All authors conducted the experiments during multiple beamtimes at BESSY II. H. A., R. S. and B. W. analyzed the data, and wrote the article. All authors have given approval to the final version of the manuscript.

## Funding sources

H. A. thanks the Egyptian Ministry of Higher Education and Ain Shams University for her PhD grant, and the Egyptian Culture Office in Berlin for support. R. S. and B. W. gratefully acknowledge financial support from the Deutsche Forschungsgemeinschaft (DFG) within the Collaborative Research Center (SFB) 1109 ‘Understanding of metal oxide/water systems at the molecular scale: structural evolution, interfaces, and dissolution’. R. S. also gratefully acknowledges an Emmy Noether Young Investigator stipend through the DFG (project SE 2253/3-1).

## Conflicts of interest

There are no conflicts to declare.

## Supplementary Material

Supplementary information

## References

[cit1] Junta-Rosso J. L., Hochella M. F. (1996). Geochim. Cosmochim. Acta.

[cit2] Wang M.-Y., Shen T., Wang M., Zhang D.-E., Tong Z.-w., Chen J. (2014). Sens. Actuators, B.

[cit3] Reufer M., Dietsch H., Gasser U., Grobety B., Hirt A. M., Malik V. K., Schurtenberger P. (2011). J. Phys.: Condens. Matter.

[cit4] Azevedo J., Seipp T., Burfeind J., Sousa C., Bentien A., Araújo J. P., Mendes A. (2016). Nano Energy.

[cit5] Ramasami A. K., Ravishankar T. N., Sureshkumar K., Reddy M. V., Chowdari B. V. R., Ramakrishnappa T., Balakrishna G. R. (2016). J. Alloys Compd..

[cit6] Dias P., Vilanova A., Lopes T., Andrade L., Mendes A. (2016). Nano Energy.

[cit7] Ketteler G., Yamamoto S., Bluhm H., Andersson K., Starr D. E., Ogletree D. F., Ogasawara H., Nilsson A., Salmeron M. (2007). J. Phys. Chem. C.

[cit8] Crumlin E. J., Bluhm H., Liu Z. (2013). J. Electron Spectrosc. Relat. Phenom..

[cit9] Karslioglu O., Nemsak S., Zegkinoglou I., Shavorskiy A., Hartl M., Salmassi F., Gullikson E. M., Ng M. L., Rameshan C., Rude B., Bianculli D., Cordones A. A., Axnanda S., Crumlin E. J., Ross P. N., Schneider C. M., Hussain Z., Liu Z., Fadley C. S., Bluhm H. (2015). Faraday Discuss..

[cit10] Wu C. H., Weatherup R. S., Salmeron M. B. (2015). Phys. Chem. Chem. Phys..

[cit11] Axnanda S., Crumlin E. J., Mao B., Rani S., Chang R., Karlsson P. G., Edwards M. O. M., Lundqvist M., Moberg R., Ross P., Hussain Z., Liu Z. (2015). Sci. Rep..

[cit12] Kolmakov A., Dikin D. A., Cote L. J., Huang J., Abyaneh M. K., Amati M., Gregoratti L., Guenther S., Kiskinova M. (2011). Nat. Nanotechnol..

[cit13] Kraus J., Reichelt R., Guenther S., Gregoratti L., Amati M., Kiskinova M., Yulaev A., Vlassiouk I., Kolmakov A. (2014). Nanoscale.

[cit14] Velasco-Velez J. J., Pfeifer V., Hävecker M., Weatherup R. S., Arrigo R., Chuang C.-H., Stotz E., Weinberg G., Salmeron M., Schlögl R., Knop-Gericke A. (2015). Angew. Chem., Int. Ed..

[cit15] Winter B. (2009). Nucl. Instrum. Methods Phys. Res., Sect. A.

[cit16] Winter B., Faubel M. (2006). Chem. Rev..

[cit17] Winter B., Weber R., Widdra W., Dittmar M., Faubel M., Hertel I. V. (2004). J. Phys. Chem. A.

[cit18] Seidel R., Thürmer S., Winter B. (2011). J. Phys. Chem. Lett..

[cit19] Faubel M., Steiner B., Toennies J. P. (1998). J. Electron Spectrosc. Relat. Phenom..

[cit20] Siefermann K. R., Liu Y. X., Lugovoy E., Link O., Faubel M., Buck U., Winter B., Abel B. (2010). Nat. Chem..

[cit21] Tang Y., Shen H., Sekiguchi K., Kurahashi N., Mizuno T., Suzuki Y. I., Suzuki T. (2010). Phys. Chem. Chem. Phys..

[cit22] Shreve A. T., Yen T. A., Neumark D. M. (2010). Chem. Phys. Lett..

[cit23] Lübcke A., Buchner F., Heine N., Hertel I. V., Schultz T. (2010). Phys. Chem. Chem. Phys..

[cit24] Congiu M., De Marco M. L., Bonomo M., Nunes-Neto O., Dini D., Graeff C. F. O. (2016). J. Nanopart. Res..

[cit25] Corbellini L., Lacroix C., Harnagea C., Korinek A., Botton G. A., Menard D., Pignolet A. (2017). Sci. Rep..

[cit26] Jubb A. M., Allen H. C. (2010). ACS Appl. Mater. Interfaces.

[cit27] Liu J., Kim Y. T., Kwon Y. U. (2015). Nanoscale Res. Lett..

[cit28] Wang W., Liang L., Johs A., Gu B. (2008). J. Mater. Chem..

[cit29] Braun A., Sivula K., Bora D. K., Zhu J., Zhang L., Graetzel M., Guo J., Constable E. C. (2012). J. Phys. Chem. C.

[cit30] Gajda-Schrantz K., Tymen S., Boudoire F., Toth R., Bora D. K., Calvet W., Gratzel M., Constable E. C., Braun A. (2013). Phys. Chem. Chem. Phys..

[cit31] Sivula K., Le Formal F., Gratzel M. (2011). ChemSusChem.

[cit32] Lemire C., Bertarione S., Zecchina A., Scarano D., Chaka A., Shaikhutdinov S., Freund H. J. (2005). Phys. Rev. Lett..

[cit33] Lad R. J., Henrich V. E. (1988). Surf. Sci..

[cit34] Kurtz R. L., Henrich V. E. (1983). Surf. Sci..

[cit35] Condon N. G., Murray P. W., Leibsle F. M., Thornton G., Lennie A. R., Vaughan D. J. (1994). Surf. Sci..

[cit36] Condon N. G., Leibsle F. M., Lennie A. R., Murray P. W., Parker T. M., Vaughan D. J., Thornton G. (1998). Surf. Sci..

[cit37] Tang Y., Qin H., Wu K., Guo Q., Guo J. (2013). Surf. Sci..

[cit38] Lanier C. H., Chiaramonti A. N., Marks L. D., Poeppelmeier K. R. (2009). Surf. Sci..

[cit39] Kiejna A., Pabisiak T. (2013). J. Phys. Chem. C.

[cit40] Kiejna A., Pabisiak T. (2012). J. Phys.: Condens. Matter.

[cit41] Alvarez-Ramírez F., Martínez-Magadán J. M., Gomes J. R. B., Illas F. (2004). Surf. Sci..

[cit42] Wang X. G., Weiss W., Shaikhutdinov S., Ritter M., Petersen M., Wagner F., Schlögl R., Scheffler M. (1998). Phys. Rev. Lett..

[cit43] Barbier A., Stierle A., Kasper N., Guittet M. J., Jupille J. (2007). Phys. Rev. B: Condens. Matter Mater. Phys..

[cit44] Parkinson G. S. (2016). Surf. Sci. Rep..

[cit45] Kuhlenbeck H., Shaikhutdinov S., Freund H. J. (2013). Chem. Rev..

[cit46] Kurtz R. L., Henrich V. E. (1987). Phys. Rev. B: Condens. Matter Mater. Phys..

[cit47] Hendewerk M., Salmeron M., Somorjai G. A. (1986). Surf. Sci..

[cit48] Todd E. C., Sherman D. M., Purton J. A. (2003). Geochim. Cosmochim. Acta.

[cit49] Todd E. C., Sherman D. M., Purton J. A. (2003). Geochim. Cosmochim. Acta.

[cit50] Morimoto T., Nagao M., Tokuda F. (1969). J. Phys. Chem..

[cit51] McCafferty E., Zettlemoyer A. C. (1971). Discuss. Faraday Soc..

[cit52] Nguyen M. T., Seriani N., Gebauer R. (2013). J. Chem. Phys..

[cit53] Yin S., Ma X., Ellis D. E. (2007). Surf. Sci..

[cit54] Pan H., Meng X., Qin G. (2014). Phys. Chem. Chem. Phys..

[cit55] von Rudorff G. F., Jakobsen R., Rosso K. M., Blumberger J. (2016). J. Phys. Chem. Lett..

[cit56] Kerisit S. (2011). Geochim. Cosmochim. Acta.

[cit57] Yamamoto S., Kendelewicz T., Newberg J. T., Ketteler G., Starr D. E., Mysak E. R., Andersson K. J., Ogasawara H., Bluhm H., Salmeron M., Brown G. E., Nilsson A. (2010). J. Phys. Chem. C.

[cit58] Spagnoli D., Gilbert B., Waychunas G. A., Banfield J. F. (2009). Geochim. Cosmochim. Acta.

[cit59] Boily J. F., Yesilbas M., Uddin M. M., Baiqing L., Trushkina Y., Salazar-Alvarez G. (2015). Langmuir.

[cit60] Thürmer S., Seidel R., Faubel M., Eberhardt W., Hemminger J. C., Bradforth S. E., Winter B. (2013). Phys. Rev. Lett..

[cit61] Suzuki Y.-I., Nishizawa K., Kurahashi N., Suzuki T. (2014). Phys. Rev. E: Stat., Nonlinear, Soft Matter Phys..

[cit62] Seidel R., Winter B. and Bradforth S. E., in Annu. Rev. Phys. Chem., ed. M. A. Johnson and T. J. Martinez, 2016, vol. 67, pp. 283–305. 10.1146/annurev-physchem-040513-103715 27023757

[cit63] Makowski M. J., Galhenage R. P., Langford J., Hemminger J. C. (2016). J. Phys. Chem. Lett..

[cit64] Brown M. A., Seidel R., Thurmer S., Faubel M., Hemminger J. C., van Bokhoven J. A., Winter B., Sterrer M. (2011). Phys. Chem. Chem. Phys..

[cit65] Lewis T., Winter B., Stern A. C., Baer M. D., Mundy C. J., Tobias D. J., Hemminger J. C. (2011). J. Phys. Chem. C.

[cit66] Seidel R., Pohl M. N., Ali H., Winter B., Aziz E. F. (2017). Rev. Sci. Instrum..

[cit67] Faubel M., Kisters T. (1989). Nature.

[cit68] Thürmer S., Seidel R., Eberhardt W., Bradforth S. E., Winter B. (2011). J. Am. Chem. Soc..

[cit69] Seidel R., Atak K., Thürmer S., Aziz E. F., Winter B. (2015). J. Phys. Chem. B.

[cit70] Lad R. J., Henrich V. E. (1989). Phys. Rev. B: Condens. Matter Mater. Phys..

[cit71] Sherman D. M. (1985). Phys. Chem. Miner..

[cit72] Yepes D., Seidel R., Winter B., Blumberger J., Jaque P. (2014). J. Phys. Chem. B.

[cit73] Radu T., Iacovita C., Benea D., Turcu R. (2017). Appl. Surf. Sci..

[cit74] Fujimori A., Saeki M., Kimizuka N., Taniguchi M., Suga S. (1986). Phys. Rev. B: Condens. Matter Mater. Phys..

[cit75] Fransson T., Harada Y., Kosugi N., Besley N. A., Winter B., Rehr J. J., Pettersson L. G. M., Nilsson A. (2016). Chem. Rev..

[cit76] Wernet P., Nordlund D., Bergmann U., Cavalleri M., Odelius M., Ogasawara H., Naslund L. A., Hirsch T. K., Ojamae L., Glatzel P., Pettersson L. G. M., Nilsson A. (2004). Science.

[cit77] Poshusta R. D., Tseng D. C., Hess A. C., McCarthy M. I. (1993). J. Phys. Chem..

[cit78] Cressey G., Henderson C. M. B., van der Laan G. (1993). Phys. Chem. Miner..

[cit79] Bora D. K., Braun A., Erat S., Ariffin A. K., Löhnert R., Sivula K., Töpfer J., Grätzel M., Manzke R., Graule T., Constable E. C. (2011). J. Phys. Chem. C.

[cit80] Wu Z. Y., Gota S., Jollet F., Pollak M., Gautier-Soyer M., Natoli C. R. (1997). Phys. Rev. B: Condens. Matter Mater. Phys..

[cit81] Pollak M., Gautier M., Thromat N., Gota S., Mackrodt W. C., Saunders V. R. (1995). Nucl. Instrum. Methods Phys. Res., Sect. B.

[cit82] Park T.-J., Sambasivan S., Fischer D. A., Yoon W.-S., Misewich J. A., Wong S. S. (2008). J. Phys. Chem. C.

[cit83] Colliex C., Manoubi T., Ortiz C. (1991). Phys. Rev. B: Condens. Matter Mater. Phys..

[cit84] de Groot F. M. F., Grioni M., Fuggle J. C., Ghijsen J., Sawatzky G. A., Petersen H. (1989). Phys. Rev. B: Condens. Matter Mater. Phys..

[cit85] Gilbert B., Frandsen C., Maxey E. R., Sherman D. M. (2009). Phys. Rev. B: Condens. Matter Mater. Phys..

[cit86] Winter B., Aziz E. F., Hergenhahn U., Faubel M., Hertel I. V. (2007). J. Chem. Phys..

[cit87] Shimizu K., Shchukarev A., Kozin P. A., Boily J. F. (2013). Langmuir.

[cit88] Shchukarev A., Boily J.-F. (2008). Surf. Interface Anal..

[cit89] Brown M. A., Winter B., Faubel M., Hemminger J. C. (2009). J. Am. Chem. Soc..

[cit90] Aziz E. F., Ottosson N., Faubel M., Hertel I. V., Winter B. (2008). Nature.

[cit91] Golnak R., Bokarev S. I., Seidel R., Xiao J., Grell G., Atak K., Unger I., Thurmer S., Aziz S. G., Kuhn O., Winter B., Aziz E. F. (2016). Sci. Rep..

[cit92] Golnak R., Xiao J., Atak K., Unger I., Seidel R., Winter B., Aziz E. F. (2016). J. Phys. Chem. A.

[cit93] Björneholm O., Nilsson A., Sandell A., Hernnas B., Martensson N. (1992). Phys. Rev. Lett..

[cit94] Nordlund D., Ogasawara H., Bluhm H., Takahashi O., Odelius M., Nagasono M. (2007). Phys. Rev. Lett..

[cit95] Campbell J. L., Papp T. (2001). At. Data Nucl. Data Tables.

[cit96] Krause M. O., Oliver J. H. (1979). J. Phys. Chem. Ref. Data.

[cit97] Core Level Spectroscopy of Solids, ed. F. de Groot and A. Kotani, CRC Press, Boca Raton, London, New York, 2008.

[cit98] Slavíček P., Kryzhevoi N., Aziz E. F., Winter B. (2016). J. Phys. Chem. Lett..

[cit99] Thürmer S., Unger I., Slavíček P., Winter B. (2013). J. Phys. Chem. C.

[cit100] Cappa C. D., Smith J. D., Messer B. M., Cohen R. C., Saykally R. J. (2007). J. Phys. Chem. A.

