# Supplementary material for: Molecular species forming at the α-Fe2O3 nanoparticle–aqueous solution interface
Source: Chem Sci. 2018 Apr 20;9(19):4511–23. doi: 10.1039/c7sc05156e (PMC5961451; doi:10.1039/c7sc05156e)
Supplement: Supplementary file 1 [file SC-009-C7SC05156E-s001.pdf]

Electronic Supplementary Material (ESI) for Chemical Science.
This journal is © The Royal Society of Chemistry 2018

Supporting Information

Molecular species forming at the α-Fe_2O_3 nanoparticle – aqueous solution interface

Hebatallah Ali,[1,2,3] Robert Seidel,[3,4] Marvin N. Pohl,[1,2] and Bernd Winter [1,*]

[1] *Fritz-Haber-Institut der Max-Planck-Gesellschaft, Faradayweg 4-6, D-14195 Berlin, Germany*

[2] *Fachbereich Physik, Freie Universität Berlin, Arnimallee 14, D-14195 Berlin, Germany*

[3] *Helmholtz-Zentrum Berlin für Materialien und Energie, Albert-Einstein-Straße 15, D-12489 Berlin, Germany*

[4] *Humboldt-Universität zu Berlin, Department of Chemistry, Brook-Taylor-Str. 2, D-12489 Berlin, Germany*

[*] *Corresponding author: winter@fhi-berlin.mpg.de*

Additional Information on: Lattice-oxygen O 1s absorption spectra from Fe_2O_3 NP (aq)

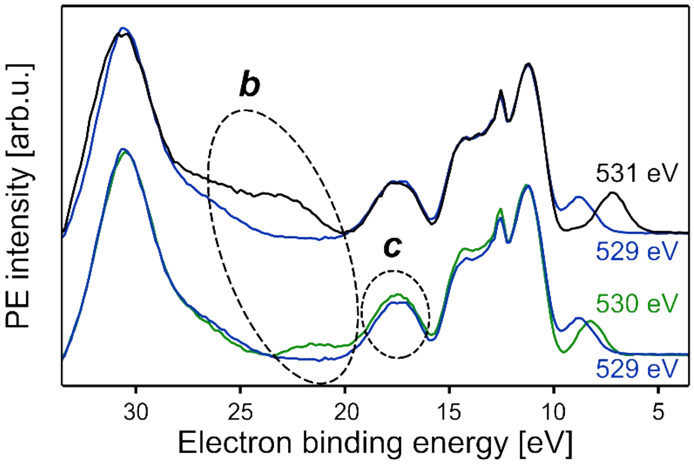

Figure SI-1 Selected RPE spectra from 5 wt% NP / 0.1 M HNO_3 aqueous solution which are taken from Figure 2A for photon energies 530.0 eV (***c*** resonance) and 531.0 eV (energy between ***c*** and ***b*** resonances). Each spectrum is presented together with the off-resonant valence PE spectrum measured at 529.0 eV. Important to notice is the slightly larger intensity in the 530-eV spectrum at 17.5 eV BE (bottom tier), which disappears shortly above the resonance (top tier). This is the same contribution that gives rise to the weak signal enhancement, labeled ***c***, in Figure 2C. All peaks appearing in the < 10 eV BE region result from water O 1s ionization by the second-order light (2x hν) which is not blocked at the beamline.

Additional Information on: Nitrate and hydroxide O 1s absorption spectra from Fe_2O_3 NP aqueous solutions

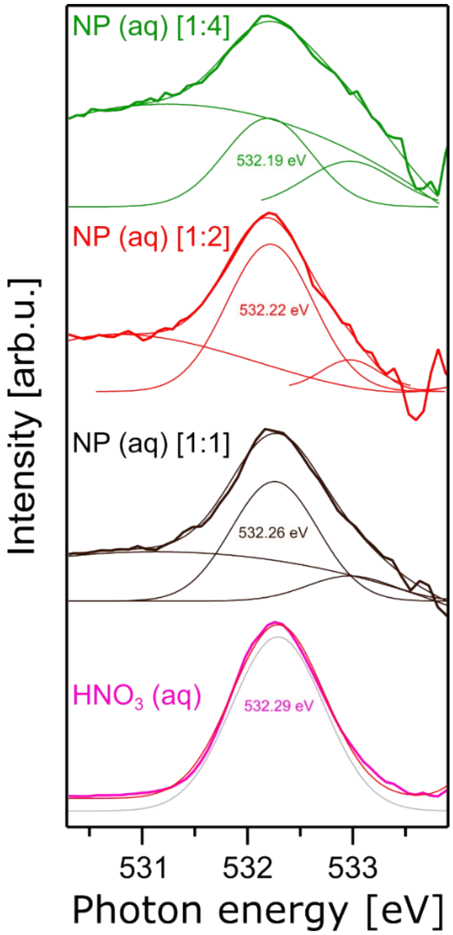

Figure SI-2 PEY-XA spectra from 0.5 M HNO_3 aqueous solution and from three NP solutions, 5 wt% NP / 0.1 M HNO_3, 10 wt% NP / 0.1 M HNO_3, and 10 wt% NP / 0.05 M HNO_3. The presented photon energy region 531-534 eV corresponds to absorption ***a*** in Figure 2B. Spectra were generated by integration of the respective RPE spectral intensities as a function of photon energy as explained for Figure 2 in the main text. In Figure SI-2 the spectra from different solutions were energy-calibrated and intensity-normalized at the O 1s liquid water pre-peak at 535.0 eV photon energy, after subtraction of the water-vapor absorption spectrum; compare Figure 3A in the manuscript. For the 5 wt% solution the ratio of available NO_3^- molecules to adsorption sites at the NP (6 nm diameter) surface is approximately 1:1. That is, the surface of the NPs is expected to be fully covered by adsorbed nitrate, and the concentration of aqueous-phase NO_3^- (aq) should be very low. Then, at 10 wt% NP concentration in 0.1 M HNO_3 and in 0.05 M HNO_3, the nitrate-to-surface site ratio decreases to 1:2 and 1:4, implying that H_2O molecules can now interact directly with the NP surface, forming OH interfacial species. Above ratios are a coarse estimate based on the total surface of the NPs in a given volume, and assuming a density of adsorption sites of 5.6 nm^{-2} which is the value reported for crystalline hematite. [E. McCafferty and A. C. Zettlemoyer, Discuss. Faraday Soc., 1971, 239.] Smaller ratios were not investigated as to avoid agglomeration of the NPs; all measurements presented here were performed from freshly prepared NP solutions.

Additional Information on: Adsorbed nitrate and hydroxide on the NP surface observed in the O 1s RPE spectra from Fe_2O_3 NP aqueous solutions

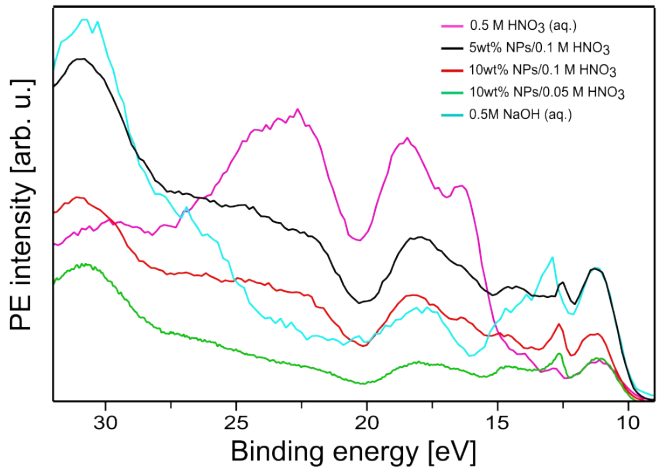

Figure SI-3 Photoelectron spectra at resonance ***a*** (532.2 eV) for the same three α-Fe_2O_3 NPs aqueous solutions considered in Figure SI-2. In addition, spectra from 0.5 M HNO_3 and 0.5 M NaOH (measured at near ***a***) aqueous solution, are shown. Unlike in Figure 4 of the manuscript spectra are shown as measured without the off-resonant photoelectron spectrum subtracted. Otherwise Figure SI-3 and Figure 4 are identical.

Additional Information on: Valence photoelectron spectra from 1 M HNO_3 aqueous solution

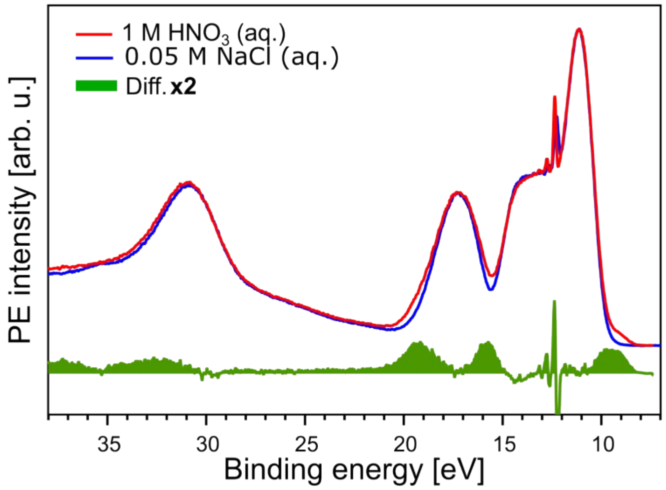

Figure SI-4 Valence photoelectron spectra from 1 M HNO_3 and from 0.05 M NaCl (which is essentially the spectrum from neat liquid water) aqueous solutions measured at 200 eV photon energy. The green curve is the difference spectrum, HNO_3 minus NaCl, and highlights the signal contributions from ionization of NO_3^- (aq). Peaks occur at 9.5, 16.0, 19.5 eV binding energy; these energies are in good agreement with computed energies. [R. D. Poshusta, D. C. Tseng, A. C. Hess and M. I. McCarthy, *J. Phys. Chem.*, 1993, **97**, 7295-7303]

Additional Information on: O 1s photoelectron spectra from Fe_2O_3 NP aqueous solution

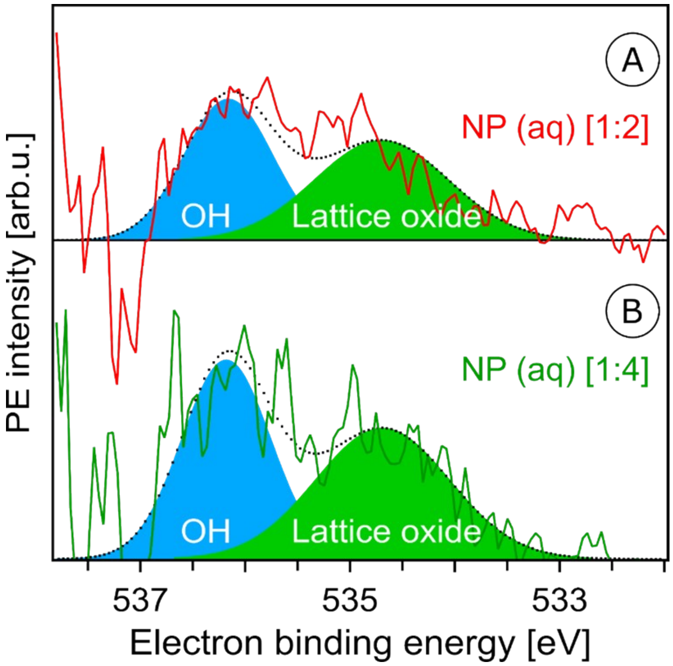

Figure SI-5 Oxygen 1s photoelectron spectra from the 10 wt% hematite NPs in 0.1 M HNO_3 aqueous solution showing the 538-532 eV binding energy region which covers the O 1s photoelectron spectra from OH^- (536.1 eV) and lattice oxide (534.7 eV). Ionization photon energy was 1200 eV. In this figure we present the raw data, whereas in Figures 5B and 5C we show the same data for 5-point binning. Each contribution is presented by a Gaussian, and the total fit is shown by the dotted curve. (A) shows results for the 10 wt% hematite NPs / 0.1 M HNO_3 aqueous solution (corresponding to [1:2]), and (B) for 10 wt% hematite NPs / 0.05 M HNO_3 aqueous solution (corresponding to [1:4]) after subtraction of the 0.05 M NaCl aqueous solution spectrum.

The table below presents the positions and areas of both the hydroxide and lattice-oxide O1s photoelectron peaks along with the respective error bars of the raw data.

NPs in 0.1 M HNO_3 aq solution	Peak [eV]	Area [arb. u.]
Lattice Oxide	534.8 ± 0.2	114.4 ± 32
OH^-	536.2 ± 0.1	91.2 ± 29

NPs in 0.05M HNO_3 aq solution	Peak [eV]	Area [arb. u.]
Lattice Oxide	534.7 ± 0.2	139.2 ± 31
OH^-	536.2 ± 0.1	140.2 ± 30
